# *Pseudomonas aeruginosa* Pyocyanin Increases Airway Mucin Biosynthesis by SIRT1-Mediated FOXA2 Inactivation

**DOI:** 10.3390/ijms27198882

**Published:** 2026-10-06

**Authors:** Woosuk Choi, Beata Kosmider, Gee W. Lau

**Affiliations:** 1 Department of Pathobiology, College of Veterinary Medicine, University of Illinois Urbana-Champaign, Urbana, IL 61802, USA; 2 Center for Inflammation and Lung Research, Department of Microbiology, Immunology and Inflammation, Lewis Katz School of Medicine, Temple University, Philadelphia, PA 19140, USA; beata.kosmider@temple.edu

**Keywords:** *Pseudomonas aeruginosa*, pyocyanin, oxidative stress, SIRT1, FOXA2

## Abstract

The potent redox-cycling *Pseudomonas aeruginosa* secondary metabolite pyocyanin induces acute and persistent oxidative stress in diseased airways. Pyocyanin promotes goblet cell hyperplasia and metaplasia and exacerbates the mucus burden. Forkhead Box A2 (FOXA2) is a key pioneer factor that governs the transition of airway basal epithelial and club cells to goblet cells by transcriptionally repressing the expression of *MUC5AC* and *MUC5B* mucin genes. FOXA2 activity is negatively regulated by Thr156 phosphorylation in various cell types. However, how post-translational modifications modulate the transcriptional repression of FOXA2 on airway mucin genes remains uncharacterized. Here, we demonstrate that the interaction between SIRT1 and FOXA2 is significantly increased within pyocyanin-exposed *MUC5AC*-expressing human bronchial epithelial cells, and in human COPD and pyocyanin-exposed mouse lungs. Pyocyanin promotes SIRT1-mediated deacetylation of FOXA2, which serves as a prerequisite for subsequent phosphorylation, inactivation, and *MUC5AC* derepression. Specifically, under pyocyanin-imposed oxidative stress, SIRT1 deacetylates FOXA2 at Lys259, which dictates phosphorylation at Thr156, leading to nuclear exclusion, ubiquitination, and proteasome-mediated degradation, initiating airway goblet cell transdifferentiation. By contrast, inactivation of SIRT1 by transient knockdown and chemical inhibition impedes pyocyanin-induced FOXA2 phosphorylation and *MUC5AC* expression. Our results uncover a novel role of SIRT1 in regulating FOXA2 expression dynamics governing airway mucus homeostasis.

## 1. Introduction

The airway mucus layer serves as a primary innate defense system, protecting the lungs by trapping and eliminating inhaled irritants and pathogens through mucociliary clearance [1]. However, pathological mucus accumulation paradoxically impairs lung function by narrowing airway lumens and limiting airflow, a hallmark of chronic respiratory diseases such as chronic obstructive pulmonary disease (COPD), cystic fibrosis (CF), non-CF bronchiectasis, and chronic bronchitis [2,3,4,5,6]. Chronic proinflammatory environments drive goblet cell hyperplasia and metaplasia, leading to airway mucus obstruction through a combination of mucus hypersecretion and impaired clearance [7,8,9]. Goblet cells maintain airway mucus homeostasis by modulating the production of gel-forming mucin glycoproteins, primarily MUC5AC and MUC5B [10,11]. The expression of both mucins is markedly upregulated under inflammatory stress and allergen exposure [10,12]. In addition, mucus-laden airways create a permissive microenvironment for chronic microbial colonization and infections, which further exacerbate mucus pathology and directly increase the mortality and morbidity in patients with muco-obstructive diseases [8,13,14,15,16].

*Pseudomonas aeruginosa* (*PA*) is a major opportunistic Gram-negative pathogen in the lungs of people with CF and advanced stages of COPD, where its presence correlates with the severity of acute exacerbations [17,18,19]. *PA* is recalcitrant to broad spectrums of antibiotics and expresses a plethora of virulence factors to neutralize host defense systems and create a microenvironment favorable to chronic infection [20,21,22]. Among the virulence factors, pyocyanin (PCN) is a potent redox-cycling secondary metabolite that induces persistent oxidative stress by continuously generating reactive oxygen/nitrogen species (ROS/RNS) [23,24,25]. While PCN-mediated ROS/RNS are damaging to competing microbiota and host cells, *PA* utilizes PCN to regulate its own metabolic homeostasis and intercellular communication, allowing it to become the predominant species over the long course of chronic infection [26,27]. In the context of respiratory infection, PCN facilitates vicious cycles between *PA* colonization and airway mucus accumulation by impeding mucociliary clearance through coordination of ciliostasis and mucus hypersecretion [24,28,29,30,31]. Previously, we have shown that PCN enhances goblet cell transdifferentiation and MUC5AC and MUC5B biosynthesis by hyperactivating EGFR-AKT/ERK1/2 and STAT6-SPDEF signaling pathways [29,32,33,34], which converge to inhibit Forkhead Box A2 (FOXA2) expression. We have shown that inhibition of FOXA2 is a critical molecular event driving airway goblet cell hyperplasia and metaplasia across diverse microbial pathogens and mammalian hosts [28,29,35,36,37].

FOXA2 is a master pioneer factor that regulates lung alveolarization and branching morphogenesis by directing respiratory epithelial cell fate [38,39]. FOXA2 depletion robustly induces goblet cell differentiation and upregulates the expression of *MUC5AC* and *MUC5B* [38,40,41]. Indeed, mice with conditional deletion of *FOXA2* driven by promoters of surfactant protein C (*Sftpc*) and club cell secretory proteins (*Scgb1a1*) ectopically develop emphysema and bronchial goblet cell hyperplasia, respectively [38]. Similarly, during airway remodeling under PCN-induced oxidative stress, EGFR ligands and type 2 helper T cell (Th2) cytokines promote FOXA2 depletion through the activation of EGFR-AKT/ERK1/2 and IL-4/IL-13 receptor-STAT6-SPDEF signaling cascades [28,29,35,36,37]. Specifically, AKT phosphorylates and inactivates FOXA2, triggering its translocation from the nucleus to the cytoplasm and subsequent proteasomal degradation [42]. However, beyond phosphorylation, the precise modulation of FOXA2 activity and stability throughout its lifecycle likely necessitates additional post-translational modifications.

Indeed, dynamic acetylation/deacetylation cycles have emerged as a critical regulator of FOXA2 function and stability. FOXA2 harbors multiple lysine residues, each of which can be selectively targeted by distinct deacetylases in a context-dependent manner [43,44,45,46]. Among members of the histone deacetylase (HDAC) superfamily, the class III deacetylase sirtuin 1 (SIRT1) triggers functional changes in FOXA2 via deacetylation. In the context of insulin signaling, SIRT1-mediated deacetylation coordinates with AKT-mediated phosphorylation at Thr156 to synergistically inactivate FOXA2 [43,44]. Specifically, SIRT1 deacetylates Lys259 on FOXA2, which triggers phosphorylation at Thr156, nuclear exclusion, and subsequent degradation. As a member of the NAD^+^-dependent sirtuin family, SIRT1 maintains cellular homeostasis by modulating various physiological responses, including oxidative stress [47]. Within the antioxidant defense system, SIRT1-mediated deacetylation activates the FOXO1/3/4 and NRF2 transcription factors, thereby upregulating the expression of antioxidant enzymes in bronchial epithelial cells [48]. Notably, the SIRT1-NRF2 axis has been shown to regulate mucus production in response to IL-13-induced inflammatory responses [49]. Although our previous findings established that PCN stimulates oxidative stress and Th2-driven inflammatory pathways [29,33,50], it remains to be elucidated whether PCN induces SIRT1 to deacetylate FOXA2, leading to downstream phosphorylation-mediated inactivation, goblet cell transdifferentiation and mucin overexpression.

In this study, we examined the impact of SIRT1-mediated deacetylation on the functional stability of FOXA2 under PCN exposure in human bronchial epithelial cells and in a mouse model of acute PCN exposure. Molecular and functional analyses demonstrate that, in the presence of PCN, SIRT1 directly deacetylates FOXA2, leading to its phosphorylation and degradation, and elevated *MUC5AC* biosynthesis. Consistent with previous studies, our findings indicate that Lys259 is a key deacetylation residue that dictates FOXA2 inactivation and degradation in PCN-exposed airway epithelial cells, indicating that SIRT1-mediated FOXA inactivation is a conserved molecular mechanism across various cell subsets.

## 2. Results

### 2.1. PCN Exposure Induces SIRT1 Protein Expression and FOXA2 Depletion in MUC5AC-Expressing Bronchial Epithelial Cells

**Figure 1:**
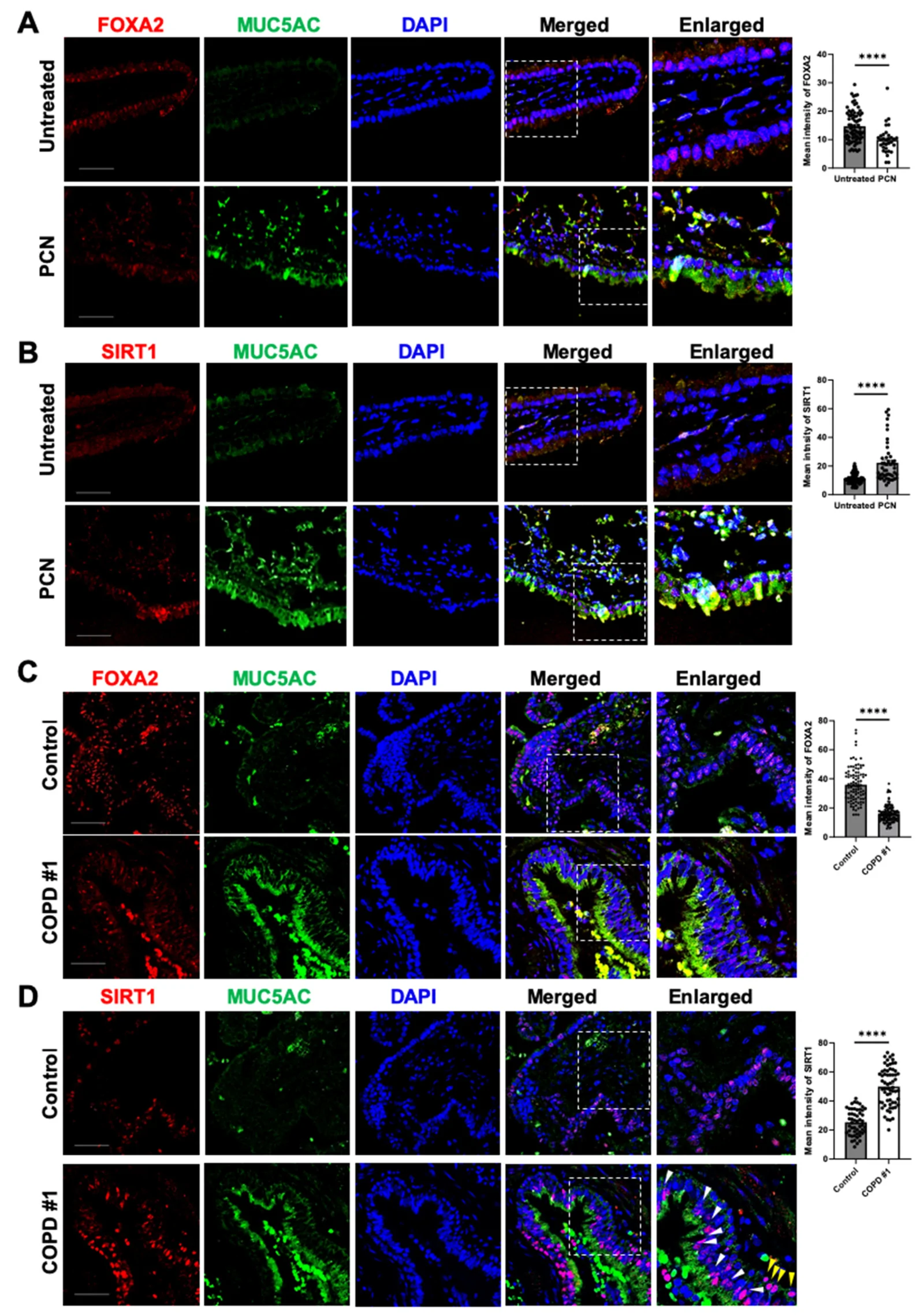
PCN induces FOXA2 depletion and SIRT1 upregulation in MUC5AC^+^ bronchial goblet cells within the lungs of PCN-exposed mice and COPD #1 patients. (**A**–**D**) Endogenous FOXA2 (red), SIRT1 (red), and MUC5AC (green) expressions in the lungs from control and PCN-exposed mice (**A**,**B**) and in human lungs from control organ donors and COPD #1 patients (**C**,**D**) were detected by immunofluorescence staining. White arrowheads indicate SIRT1^high^ goblet cells, and yellow arrowheads show SIRT1^high^ basal epithelial cells (**D**). Each target was probed with a target-specific antibody and visualized by fluorescence-conjugated secondary antibody (Alexa Fluor 488 (green) or Alexa Fluor 594 (red)). Nuclei were visualized by DAPI. Regions of interest (ROI) indicated in boxes were enlarged. The intensity of FOXA2 and SIRT1 was measured by ImageJ. Statistics were analyzed by the unpaired Welch’s *t*-test. Values are the mean ± S.E.M.; **** *p* < 0.0001.

Previous studies in insulin signaling and cellular metabolism have shown that phosphorylation at Thr156 is critical for FOXA2 inactivation and subsequent depletion [42,43]. Furthermore, SIRT1-mediated deacetylation precedes FOXA2 phosphorylation and degradation [43,44]. However, it is not known whether deacetylation by SIRT1 leads to subsequent phosphorylation and inactivation of FOXA2 and initiation of airway goblet cell transdifferentiation during PCN-mediated persistent oxidative stresses. To verify crosstalk between FOXA2 and SIRT1 in goblet cell transdifferentiation, we first examined FOXA2 and SIRT1 protein expression in MUC5AC-producing bronchial epithelial cells in the lungs of PCN-exposed mice and COPD patients. PCN-exposed mouse airways showed increased expression of nuclear-localized SIRT1 and a corresponding decrease in nuclear FOXA2 in bronchial epithelial cells (Figure 1A,B). Similarly, in COPD lungs, nuclear FOXA2 abundance was markedly depleted in majority of goblet cells (Figure 1C). Concurrently, a distinct population of SIRT1^high^MUC5AC^+^ goblet cells lined in the airway epithelium, although not all the MUC5AC^+^ goblet cells exhibited increased expression of SIRT1 (Figure 1D). Notably, SIRT1 expression was markedly increased in both goblet cells and basal epithelial cells in COPD lungs compared to control lungs (Figure 1D).

**Figure 2:**
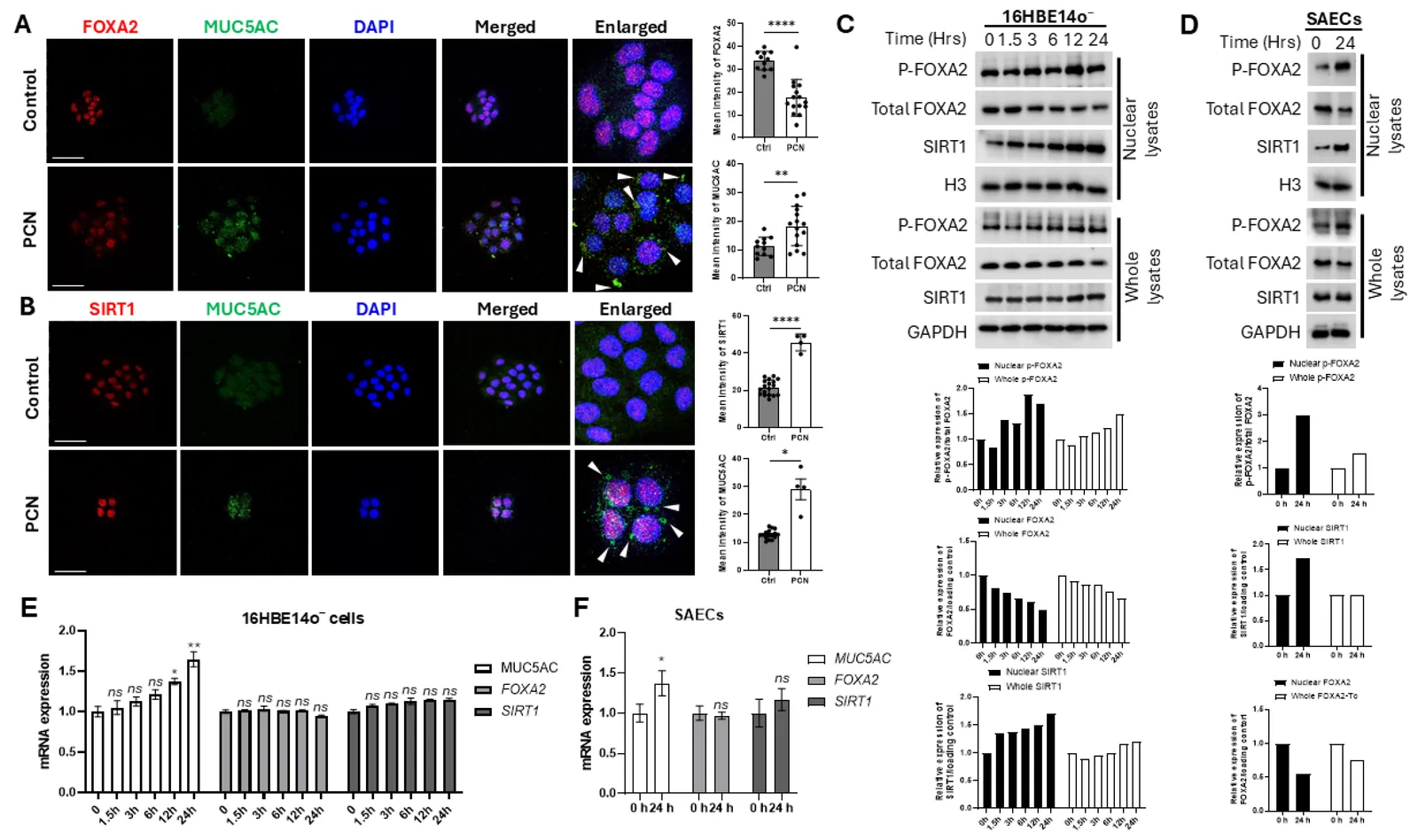
PCN induces FOXA2 depletion and SIRT1 upregulation in MUC5AC-expressing human bronchial cells. (**A**,**B**) 16HBE14o^−^ cells were exposed to 5 µg/mL PCN for 24 h. FOXA2, SIRT1, and MUC5AC expressions were probed with target-specific antibodies and subsequently visualized by fluorescence-conjugated secondary antibodies (Alexa Fluor 488 (green) and 594 (red)). Arrowheads depict MUC5AC depositions in the cells. Nuclei were counterstained with DAPI. The fluorescence intensities of FOXA2 (red), SIRT1 (red), and MUC5AC (green) were quantified using ImageJ. Statistical significance for (**A**,**B**) was analyzed by Welch’s *t*-test. (**C**–**E**) 16HBE14o^−^ cells were exposed to 5 µg/mL PCN in a time-dependent manner. SAECs were exposed to 5 µg/mL PCN for 24 h. (**C**) Expression levels of phosphorylated FOXA2 (p-FOXA2), total FOXA2, and SIRT1 in nuclear and whole-cell lysates of 16HBE14o^−^ cells were assessed by Western blot analysis. (**D**) Expression levels of phosphorylated FOXA2 (p-FOXA2), total FOXA2, and SIRT1 in nuclear and whole-cell lysates of SAECs were assessed by Western blot analysis. Densitometric quantification of p-FOXA2, total-FOXA2, and SIRT1 was performed using ImageJ, with nuclear and whole-cell lysate fractions normalized to histone H3 and GAPDH, respectively. (**E**) *MUC5AC*, *FOXA2*, and *SIRT1* mRNA expressions in 16HBE14o^−^ cells treated with PCN in a time-dependent manner were measured by real-time qPCR. (**F**) *MUC5AC*, *FOXA2*, and *SIRT1* mRNA expressions in SAECs treated with PCN for 24 h were measured by real-time qPCR (*n =* 3). Data in (**C**–**E**) were analyzed by one-way ANOVA with Dunnett’s post hoc test across 6 samples together with the unpaired Student’s *t*-test for comparing the control group with each of the PCN-treated groups. Data in (**F**) were analyzed by the unpaired Student’s *t*-test. Values are the mean ± S.E.M.; * *p* < 0.05, ** *p* < 0.01, **** *p* < 0.0001, ns = not significant. *n* = 3.

We next examined the expression of FOXA2 and SIRT1in PCN-exposed human bronchial epithelial 16HBE14o^−^ cells and primary human small airway epithelial cells (SAECs). Consistent with the results in mouse and human COPD lungs, immunocytochemistry staining of PCN-exposed 16HBE14o^−^ showed that the nuclear localization of SIRT1 was significantly increased in MUC5AC-expressing cells (indicated by arrow heads), in contrast to a decrease in nuclear FOXA2 levels (Figure 2A,B). To confirm the subcellular localization and protein modification of FOXA2 by PCN, phosphorylated and total FOXA2 protein levels in nuclear and whole-cell lysates were quantified. Relative FOXA2 phosphorylation levels were markedly increased in PCN-exposed 16HBE14o^−^ cells in a time-dependent manner, while the protein abundance of total FOXA2 was conversely reduced over time (Figure 2C). Similar to 16HBE14o^−^ cells, FOXA2 was increasingly phosphorylated and depleted by PCN exposure in SAECs (Figure 2D). In contrast to FOXA2, nuclear SIRT1 protein abundance increased markedly in PCN-exposed 16HBE14o^−^ cells and SAECs, while SIRT1 protein levels in whole-cell lysates were marginally increased or unchanged (Figure 2C,D). FOXA2 phosphorylation levels and protein abundance in both nuclear and whole-cell lysates showed a similar trend of changes in PCN-exposed 16HBE14o^−^ cells and SAECs (Figure 2C,D), suggesting that the results obtained in the SV40-transformed 16HBE14o^−^ cells reliably recapitulate those in the SAECs.

Intriguingly, *FOXA2* and *SIRT1* mRNA expression were not significantly modulated by PCN exposure in 16HBE14o^−^ cells and SAECs, while *MUC5AC* levels were significantly elevated (Figure 2E,F). These findings suggest that changes in nuclear FOXA2 and SIRT1 protein levels are regulated post-transcriptionally. Collectively, these aforementioned data suggest that nuclear translocation allows SIRT1 to enhance its regulatory effects on nuclear targets, including FOXA2, under conditions of PCN- and disease-associated oxidative stress.

### 2.2. PCN Modulates Physical Interaction Between FOXA2 and SIRT1 in MUC5AC-Expressing Goblet Cells

**Figure 3:**
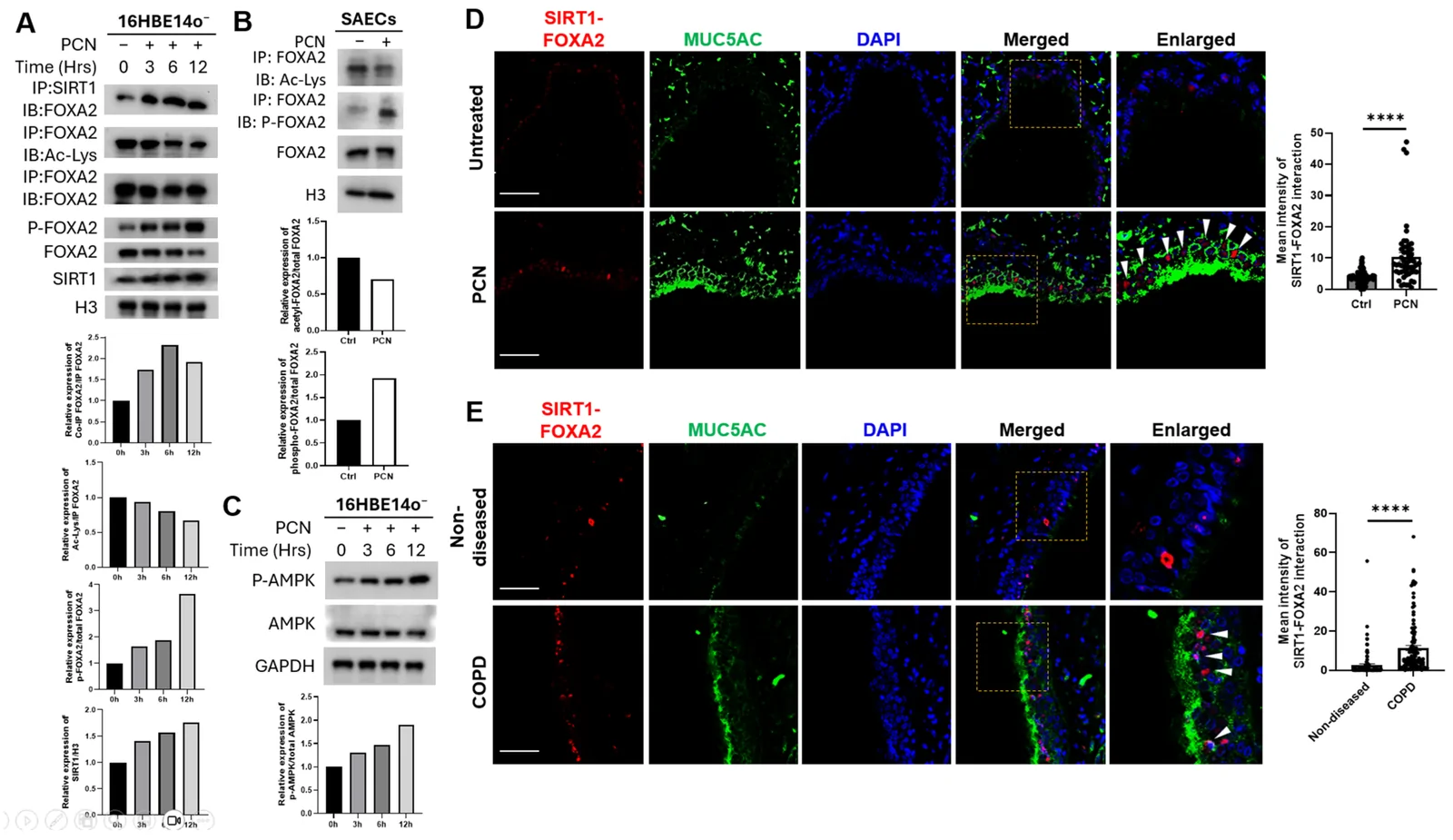
PCN induces physical interaction between SIRT1 and FOXA2 as well as FOXA2 post-translational modifications. (**A**) Changes in interaction between FOXA2 and SIRT1 as well as FOXA2 acetylation in 16HBE14o^−^ cells exposed to 5 µg/mL PCN in a time-dependent manner. For co-IP analysis of the SIRT1-FOXA2 complex, nuclear protein lysates were immunoprecipitated using an anti-SIRT1 antibody and probed for FOXA2 via Western blot analysis. FOXA2 acetylation levels were examined by FOXA2 pulldown and subsequent Western blotting by anti-acetyl lysine antibody. Nuclear p-FOXA2, FOXA2 and SIRT1 levels were monitored using histone H3 as a loading control. Because the levels of nuclear FOXA2 were depleted following PCN exposure, the relative levels of SIRT1-bound FOXA2 (Panel 1 of the Western blots) and acetylated FOXA2 (Panel 2 of the Western blots) were normalized to total IP pulldown FOXA2 (Panel 3 of the Western blots). (**B**) Changes in FOXA2 acetylation and phosphorylation in SAEC exposed to 5 µg/mL PCN for 6 h. (**C**) AMPK phosphorylation levels under PCN exposure in a time-dependent manner in 16HBE14o^−^ cells. FOXA2 in nuclear protein lysates was pulled down with an anti-FOXA2 antibody and probed with anti-acetyl lysine and anti-phospho-FOXA2 T156 antibodies. Histone H3 is used as a loading control. (**D,E**) Protein proximity ligation assay evaluating the interaction between SIRT1 and FOXA2 in the lung sections from control and PCN-exposed mice (**D**), as well as in the human lung sections of non-diseased control and COPD patients (**E**). Amplified signals of SIRT1 and FOXA2 interaction were excited at 594 nm and visualized as red fluorescence. MUC5AC expression was detected using Alexa Fluor 488-conjugated MUC5AC antibody (green). Nuclei were DAPI-stained (blue). Regions of interest (ROI) indicated in dash-lined boxes were enlarged. The proximity labeling signals were quantified by ImageJ. Statistical significance was analyzed by Welch’s *t*-test. Values are the mean ± S.E.M.; **** *p* < 0.0001.

Previous studies have demonstrated that SIRT1-mediated deacetylation inactivates FOXA2, leading to nuclear exclusion [43,44]. Because PCN induces a negative correlation between nuclear FOXA2 and SIRT1 protein levels, we hypothesized that nuclear translocation of SIRT1 promotes nuclear FOXA2 deacetylation under conditions of PCN-induced oxidative stress. To determine whether PCN modulates the physical interaction between FOXA2 and SIRT1, a protein co-IP experiment was conducted. Time-dependent PCN exposure enhanced the interactions between SIRT1 and FOXA2 in 16HBE14o^−^ cells, while FOXA2 acetylation levels were decreased (Figure 3A). In the same course of PCN treatment, nuclear phospho-FOXA2 (Thr156) and SIRT1 levels were elevated, while nuclear FOXA2 protein levels were diminished (Figure 3A). Consistent with 16HBE14o^−^ cells, PCN exposure decreased FOXA2 acetylation levels while its phosphorylation at Thr156 was significantly increased in SAECs (Figure 3B).

Next, we examined how SIRT1 was activated under PCN exposure. Previous studies have demonstrated that PCN causes ATP deprivation in host cells [51]. In response to an increase in the AMP:ATP ratio, AMP-activated protein kinase (AMPK), a master regulator of energy homeostasis, promotes NAD^+^ restoration, an essential substrate for SIRT1 activation [52]. Following PCN exposure, AMPK phosphorylation gradually increased in a time-dependent manner (Figure 3C). These in vitro findings suggest that PCN-induced ATP deprivation activates AMPK and subsequently promotes SIRT1-mediated deacetylation and inactivation of FOXA2. Notably, by using the Duolink^®^ Proximity Ligation Assay, we confirmed that the interaction between SIRT1 and FOXA2 was significantly increased in MUC5AC-expressing bronchial goblet cells of PCN-exposed mice as well as of COPD patients, compared to non-diseased control groups (arrow heads indicated in Figure 3D,E). These data suggest that SIRT1 mediates *MUC5AC* overexpression by inactivating FOXA2 through deacetylation.

### 2.3. SIRT1-Mediated FOXA2 Deacetylation Promotes MUC5AC Expression in PCN-Exposed Bronchial Epithelial Cells

**Figure 4:**
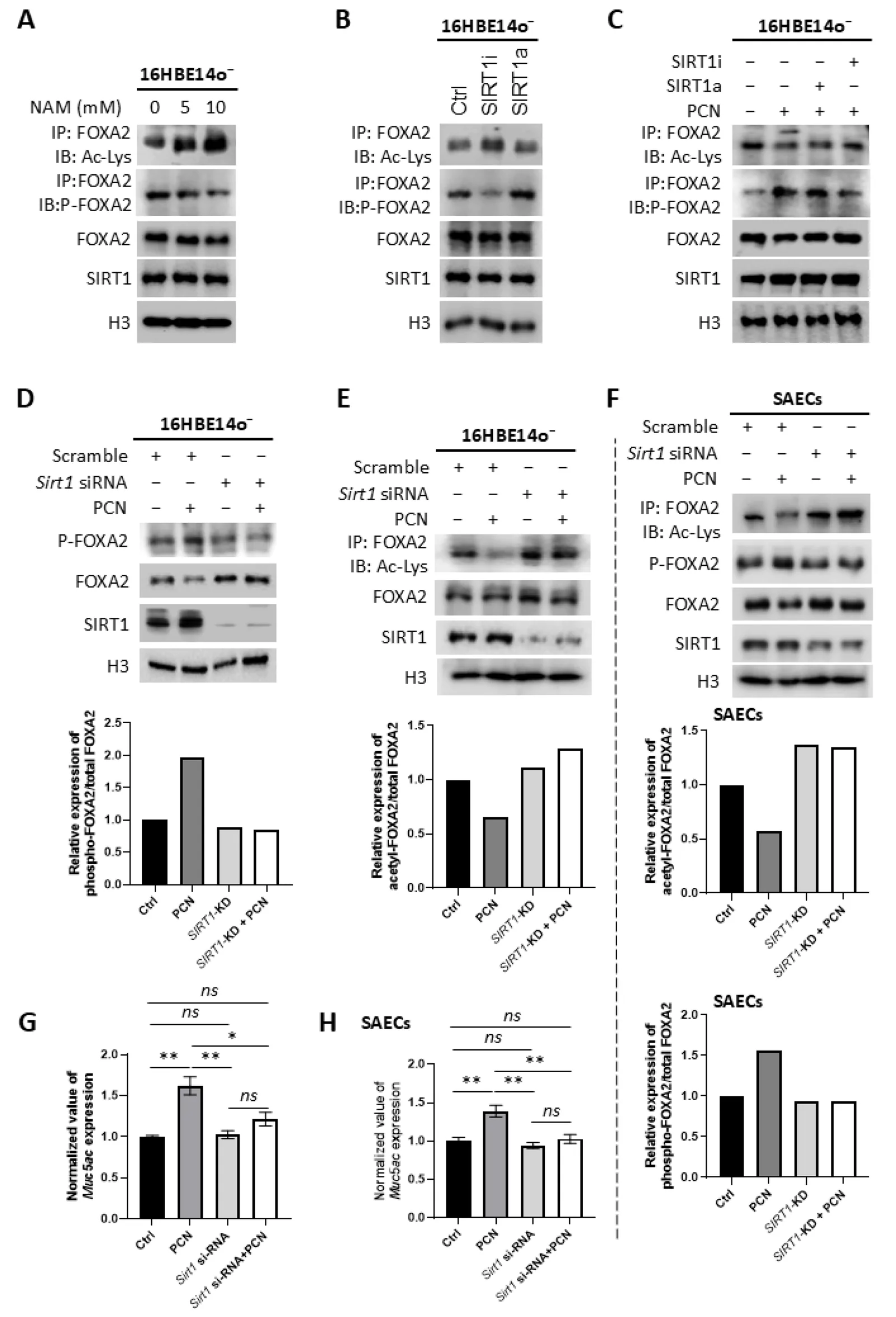
SIRT1 induces FOXA2 deacetylation, phosphorylation, nuclear exclusion, and degradation in PCN-exposed 16HBE14o^−^ cells. (**A**,**B**) Immunoprecipitation and Western blot analysis of 16HBE14o^−^ cells treated with 5 and 10 mM of the pan-sirtuin inhibitor nicotinamide (NAM) (**A**), or with 5 µM of the SIRT1-specific inhibitor EX-527 (SIRT1i) and 5 µM of the SIRT1-specific agonist SRT1720 (SIRT1a), respectively (**B**), for 24 h. Acetylated FOXA2 was immunoprecipitated from the nuclear lysates and immunoblotted with an anti-acetyl-lysine antibody. Total nuclear FOXA2 and SIRT1 levels were also assessed from nuclear lysates, with histone H3 as the loading control. (**C**) 16HBE14o^−^ cells were pretreated with the SIRT1-specific inhibitor EX-527 (SIRT1i, 5 µM) or the SIRT1-specific agonist SRT17720 (SIRT1a, 5 µM) for 3 h prior to PCN exposure for 6 h. FOXA2 was immunoprecipitated from the nuclear lysates to assess acetylation levels. Total nuclear FOXA2 and SIRT1 expression levels were analyzed by Western blot analysis with histone H3 as the loading control. (**D**,**E**) 16HBE14o^−^ cells were transfected with scrambled or *SIRT1* siRNA, followed by exposure to PCN (5 µg/mL) for 24 h. (**D**) Western blot analysis of nuclear phospho-FOXA2, FOXA2, and SIRT1. (**E**) Changes in acetylated FOXA2 levels in vehicle and *SIRT1* knockdown 16HBE14o^−^ cells with or without PCN exposure. Histone H3 was used as a loading control. Densitometric quantification of phospho-FOXA2, FOXA2, and histone H3 was performed using ImageJ. Relative phospho-FOXA2 levels over FOXA2 were calculated and normalized to the control. (**F**) Acetylation and phosphorylation levels of FOXA2 in vehicle and *SIRT1* knockdown SAECs. Nuclear FOXA2 was immunoprecipitated with an anti-FOXA2 antibody and analyzed for acetylation and T156 phosphorylation by Western blot analysis. Histone H3 was used as a loading control. Relative acetylated-FOXA2 levels over FOXA2 were calculated and normalized to control. (**G**,**H**) Real-time qPCR analysis of *MUC5AC* mRNA expression in *SIRT1* knockdown 16HBE14o^−^ cells (**G**) and SAECs (**H**) in the presence or absence of PCN. Data represent mean ± S.E.M. of n = 3. Statistical significance was determined by the two-way ANOVA test followed by Tukey’s post hoc test for multiple comparisons. * *p* < 0.05, ** *p* < 0.01, ns = not significant.

To determine whether SIRT1 deacetylates FOXA2, we treated 16HBE14o^−^ cells with nicotinamide (NAM), a pan-inhibitor of sirtuins. FOXA2 acetylation levels were increased by NAM in a dose-dependent manner, whereas FOXA2 phosphorylation at Thr156 was decreased during the same course of NAM treatment (Figure 4A). Next, we examined whether SIRT1 specifically regulates FOXA2 deacetylation. FOXA2 acetylation levels were assessed in 16HBE14o^−^ cells treated independently with SIRT1-specific inhibitor EX-527 (SIRT1i) or agonist SRT1720 (SIRT1a). FOXA2 acetylation levels were elevated in 16HBE14o^−^ cells treated with SIRT1i and decreased under SIRT1a treatment (Figure 4B). Conversely, FOXA2 phosphorylation was reduced by SIRT1i treatment and increased by SIRT1a treatment, suggesting that SIRT1-mediated deacetylation promotes FOXA2 phosphorylation and degradation. Based on the regulatory effects of SIRT1 on FOXA2 acetylation, we further examined whether SIRT1 activation and inhibition modulate FOXA2 acetylation levels in PCN-exposed 16HBE14o^−^ cells. PCN-induced FOXA2 deacetylation and phosphorylation were mitigated by SIRT1i pretreatment. In contrast, SIRT1a pretreatment did not induce additional changes in FOXA2 acetylation and phosphorylation levels in PCN-exposed 16HBE14o^−^ cells (Figure 4C).

Since SIRT1 inhibition by EX-527 impeded PCN-induced FOXA2 deacetylation, we investigated whether *SIRT1* gene knockdown would prevent the depletion of nuclear FOXA2 by modulating its acetylation and phosphorylation levels. PCN promoted FOXA2 phosphorylation and deacetylation in 16HBE14o^−^ cells transfected with scrambled siRNA, whereas *SIRT1* knockdown suppressed FOXA2 phosphorylation and deacetylation (Figure 4D,E). Moreover, *SIRT1* knockdown mitigated PCN-induced nuclear exclusion of FOXA2 (Figure 4D). Similar to 16HBE14o^−^ cells, *SIRT1* knockdown suppressed PCN-induced FOXA2 phosphorylation and deacetylation as well as nuclear FOXA2 depletion in SAECs (Figure 4F). Concomitantly, PCN-induced *MUC5AC* mRNA expression was decreased by *SIRT1* knockdown in 16HBE14o^−^ cells and SAECs (Figure 4G,H), suggesting that SIRT1-mediated deacetylation promotes FOXA2 inactivation and attenuates transcriptional repressive function against *MUC5AC* expression.

### 2.4. Lys259 Deacetylation Is Required for FOXA2 Inactivation

**Figure 5:**
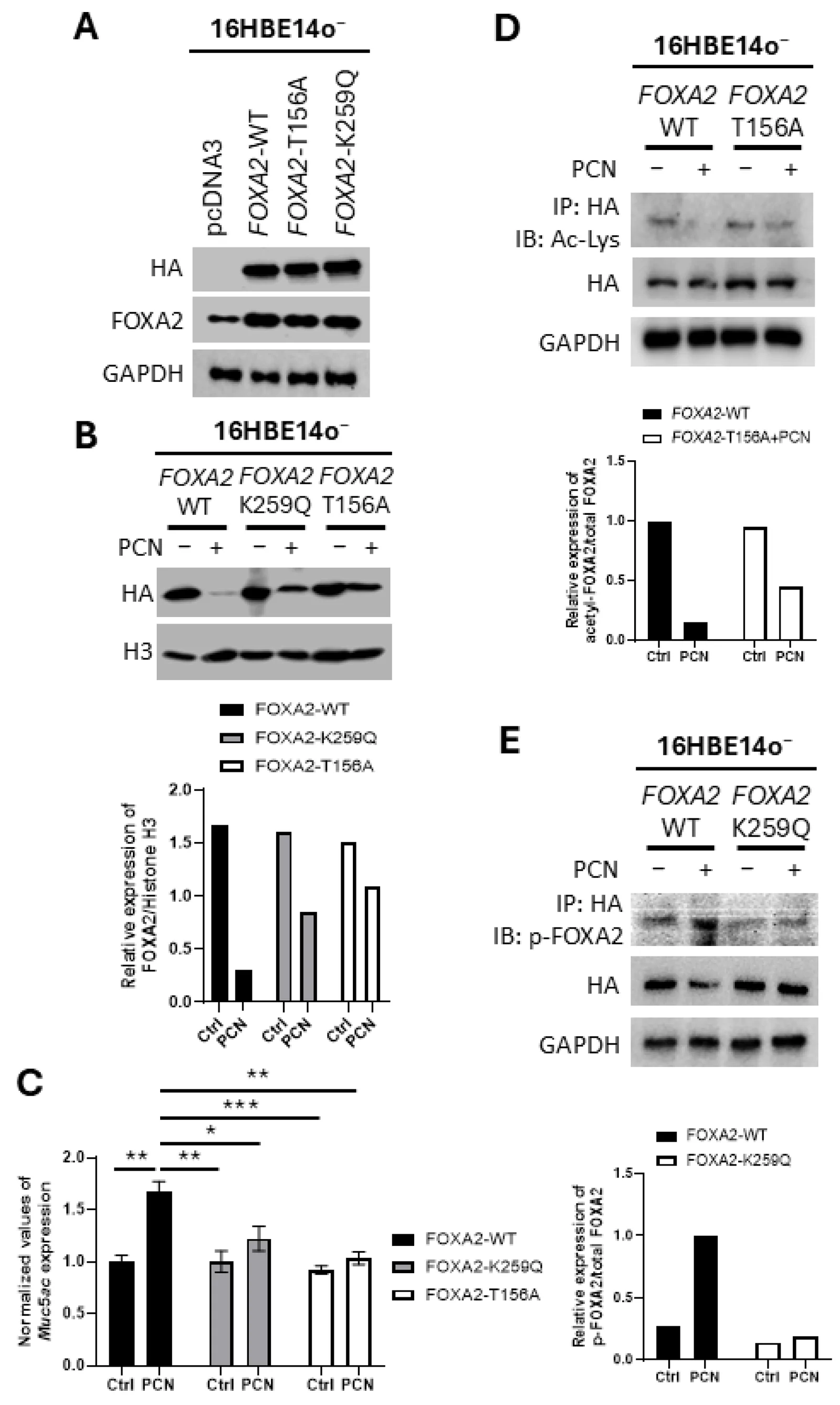
Acetylation mimicking FOXA2-K259Q and phosphorylation-deficient FOXA2-T156A mutations prevent PCN-dependent FOXA2 depletion and *MUC5AC* induction. (**A**) Western blot analysis of 16HBE14o^−^ cells transfected with HA-tagged recombinant FOXA2-WT, FOXA2-K259Q, and FOXA2-T156A. The expression of FOXA2 recombinants was detected by an anti-HA antibody, with endogenous FOXA2 and GAPDH used as an internal readout and loading control, respectively. (**B**) Nuclear protein abundances of FOXA2-WT, FOXA2-K259Q, and FOXA2-T156A during a 24 h exposure to PCN (5 µg/mL). Relative expression of FOXA2 recombinants was normalized to H3 and quantified by ImageJ. (**C**) qPCR analysis of PCN-induced *MUC5AC* mRNA in 16HBE14o^−^ cells transfected with the indicated FOXA2 recombinant plasmids. (**D**) Evaluation of Thr156 phosphorylation levels of FOXA-WT and FOXA2-K259Q in the presence of PCN. Following 24 h exposure to 5 µg/mL PCN, recombinant HA-tagged FOXA2 proteins were immunoprecipitated from whole-cell lysates with anti-HA antibody and probed with anti-phospho-FOXA2 (Thr156) antibody. Densitometric quantification of phospho-FOXA2 was conducted using ImageJ and normalized to total HA-FOXA2 levels. (**E**) Acetylation levels of FOXA2-WT and FOXA2-T156A with or without PCN exposure. Recombinant HA-tagged FOXA2 proteins were immunoprecipitated from whole-cell lysates with anti-HA antibody and probed with anti-acetyl lysine antibody. Densitometric quantification of acetyl-FOXA2 was conducted using ImageJ and normalized to total HA-FOXA2 levels. Data represent mean ± S.E.M. (n = 3). Statistical analysis was performed using two-way ANOVA followed by Tukey’s post hoc test for multiple comparison (* *p* < 0.05, ** *p* < 0.01, *** *p* < 0.001). Non-significant (ns) comparisons are omitted for clarity.

Previous studies in insulin signaling have shown that FOXA2 is inactivated by SIRT1 through deacetylation on amino acid Lys259. FOXA2-K259Q, an acetylation mimicking mutation that is resistant to deacetylation, as well as FOXA2-T156A, a phosphorylation-deficient mutation, prevent SIRT1-mediated inactivation during insulin signaling [43,44]. Because published studies and our current data suggest the notion that the regulatory mechanisms of FOXA2 turnover are conserved across various cell types, including bronchial epithelial cells, we examined whether acetylation of Lys259 regulates FOXA2 activities and nuclear localization. We transiently overexpressed HA-tagged wild-type FOXA2 (FOXA2-WT), FOXA2-K259Q, and FOXA2-T156A recombinants in 16HBE14o^−^ cells by electroporation. In the absence of PCN exposure, FOXA2-WT, FOXA2-K259Q, and FOXA2 T156A were expressed in higher levels than the pcDNA3 empty vector control (Figure 5A). Note that 16HBE14o^−^ cells harboring the pcDNA3 empty vector expressed low levels of endogenous FOXA2 when probed with an anti-FOXA2 antibody. Nuclear protein lysates from the transfected cells exposed to PCN showed that FOXA2-WT was depleted, whereas the expression of FOXA2-K259Q and FOXA2-T156A was retained at higher levels in the nuclei (Figure 5B). We further assessed whether the nuclear localization levels of recombinant FOXA2 protein would alter the transcription of the *MUC5AC* gene by real-time qPCR. When compared to 16HBE14o^−^ cells expressing FOXA2-WT, PCN-mediated *MUC5AC* induction was attenuated in the cells expressing FOXA2-K259Q and FOXA2-T156A (Figure 5C). Because the FOXA2-K259Q mutant remained active and localized to the nucleus, mimicking the behavior of the FOXA2-T156A mutant (Figure 5B), we investigated whether the FOXA2 acetylation at Lys259 impacted phosphorylation at Thr156. While PCN enhanced FOXA2-WT phosphorylation at Thr156, Thr156 phosphorylation in the FOXA2-K259Q acetylation mimicking mutation was significantly suppressed (Figure 5D). We further examined whether FOXA2-T156A phosphorylation-deficient mutation modulates FOXA2 acetylation levels under PCN exposure. Compared to FOXA2-WT, FOXA2-T156A was more resistant to deacetylation under PCN exposure (Figure 5E). These data implicate Lys259 as one of the key acetylation residues for sustaining FOXA2 activity.

**Figure 6:**
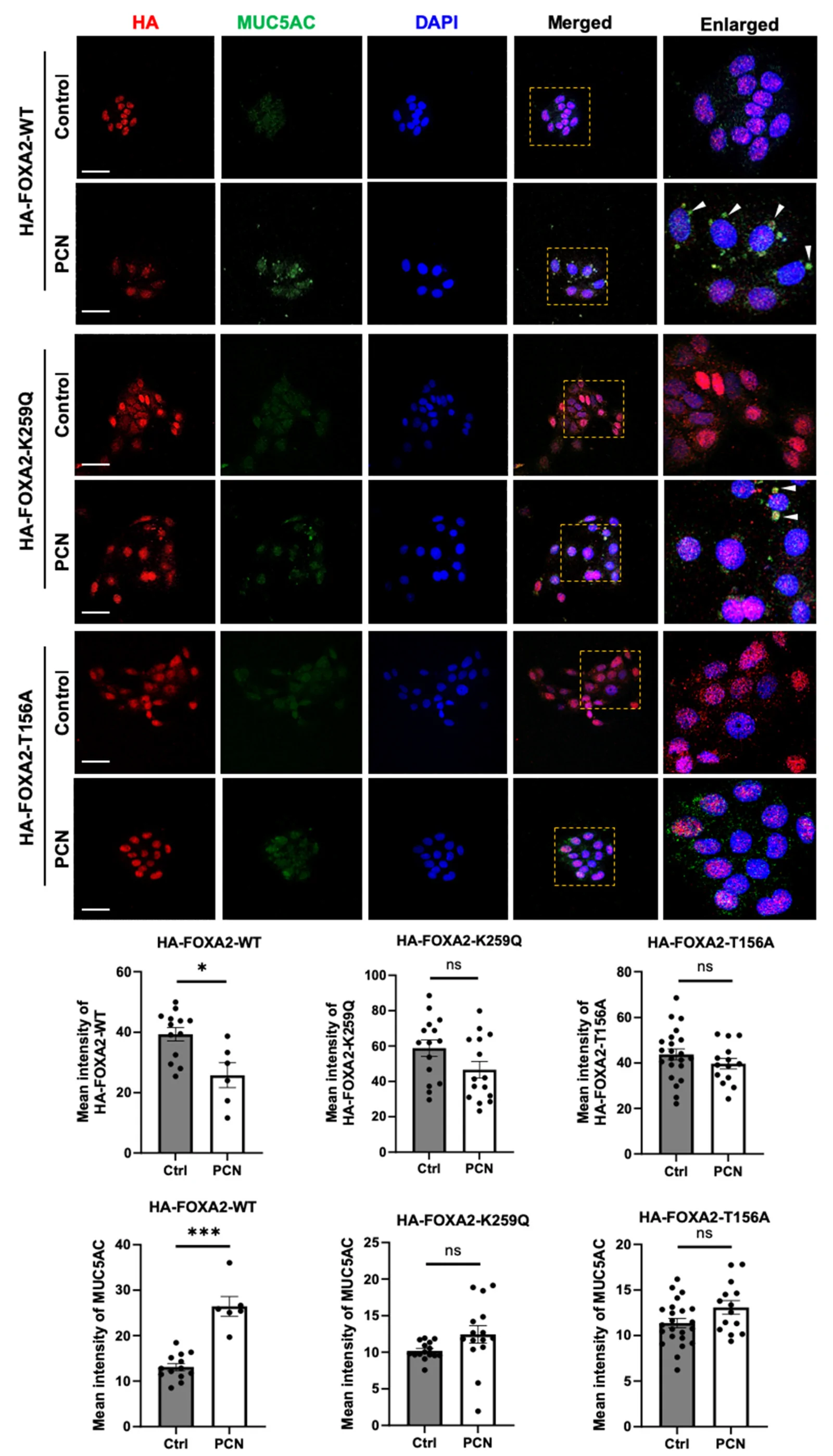
FOXA2-K259Q and FOXA2-T156A mutants are more resistant to PCN-induced nuclear exclusion and in suppressing MUC5AC expression. 16HBE14o^−^ cells were transfected with HA-tagged-FOXA2-WT, HA-FOXA2-K259Q, or HA-FOXA2-T156A recombinant plasmids, and treated with 5 µg/mL PCN for 24 h. The expression and localization of HA-FOXA2 and MUC5AC were visualized using Alexa Fluor 594 (red) and 488 (green) secondary antibodies, respectively. Arrowheads indicate intracellular MUC5AC depositions in the cells. Regions of interest (ROI) indicated in dash-lined boxes were enlarged. The fluorescence intensities of HA-tagged FOXA2 and MUC5AC were quantified using ImageJ. Statistical analysis was performed with the Welch’s *t*-test. Values are the mean ± S.E.M.; * *p* < 0.05, *** *p* < 0.001, ns = not significant.

Next, we examined the dynamics of various FOXA2 recombinants and MUC5AC in PCN-exposed 16HBE14o^−^ cells by immunofluorescence imaging (Figure 6). PCN significantly depleted nuclear FOXA2-WT, accompanied by increased MUC5AC production in 16HBE14o^−^ cells (Figure 6, indicated as arrow heads). In contrast, the expression of FOXA2-K259Q and FOXA2-T156A recombinants remained in nuclei, with a corresponding decrease in MUC5AC expression (Figure 6). These data suggest that FOXA2 deacetylation at Lys259, in coordination with subsequent phosphorylation at Thr156, is responsible for nuclear exclusion and transcription of *MUC5AC* in bronchial epithelial cells. Collectively, these results suggest that acetylation/deacetylation at Lys259 and phosphorylation at T156A modulate the transcriptional repression activity of FOXA2 on the *MUC5AC* gene.

### 2.5. Lys259 Deacetylation Synergizes with Thr156 Phosphorylation to Promote Proteasome-Mediated FOXA2 Degradation

**Figure 7:**
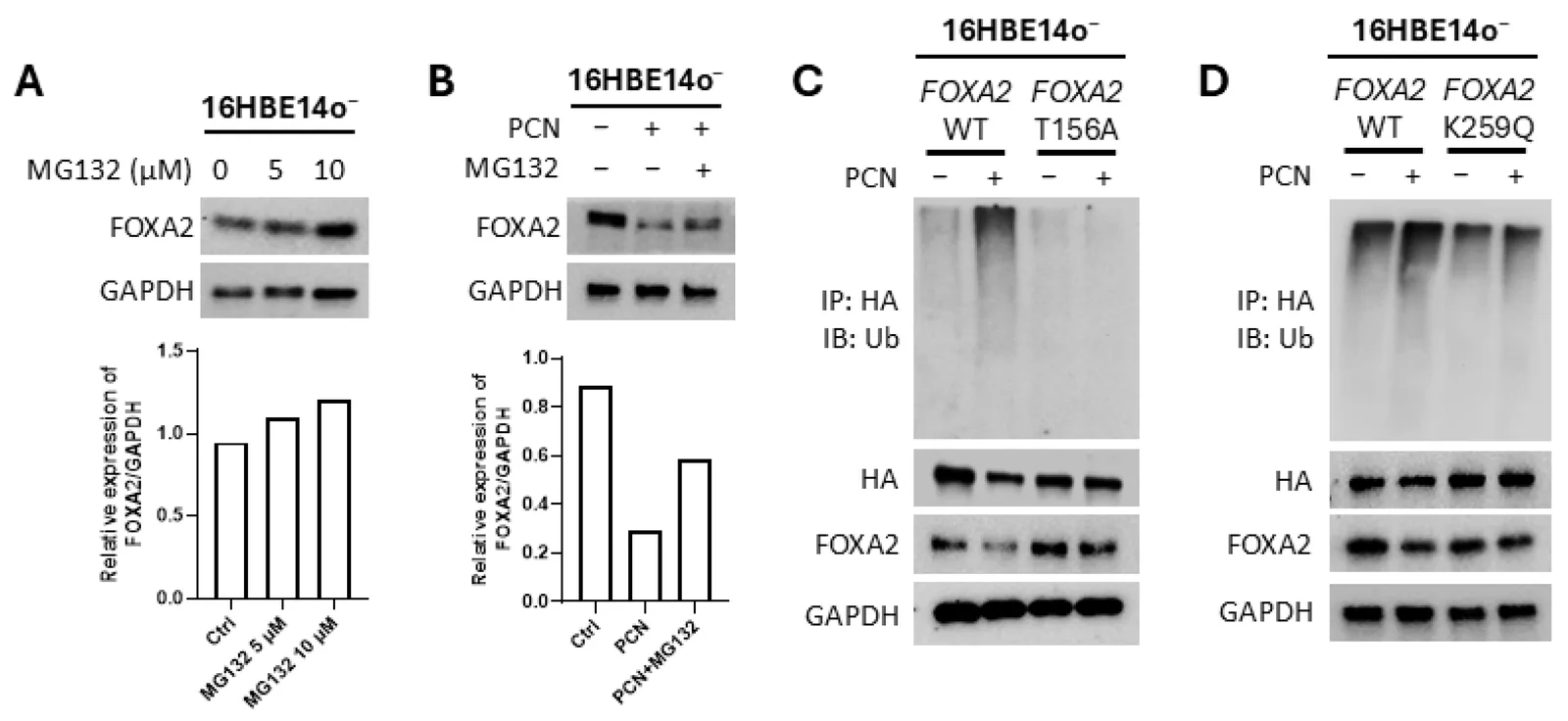
Acetylation mimicking FOXA2-K259Q mutation attenuates phosphorylation, ubiquitination, and degradation of FOXA2. (**A**,**B**) Assessment of FOXA2 degradation by 26S proteasome in the presence or absence of PCN. (**A**) 16HBE14o^−^ cells were incubated with 5 and 10 µM of the 26S proteasome MG132 for 24 h in the absence of PCN. (**B**) 16HBE14o^−^ cells were pretreated with 10 µM of MG132 for 3 h prior to 24 h exposure to 5 µg/mL PCN. Relative expression of total FOXA2 was quantified using ImageJ and normalized to GAPDH. (**C**) Analysis of PCN-induced ubiquitination levels of HA-tagged FOXA2-WT and FOXA2-K259Q. Following 24 h exposure to 5 µg/mL PCN, HA-tagged FOXA2 recombinants were immunoprecipitated from whole-cell lysates with an anti-HA antibody and probed with an anti-ubiquitin antibody. (**D**) PCN-mediated ubiquitination of FOXA2-WT and FOXA2-K259Q. HA-tagged FOXA2 proteins were immunoprecipitated from whole-cell lysates with an anti-HA antibody and probed with an anti-ubiquitin antibody. Total FOXA2 and GAPDH were used as the internal readout and loading control, respectively.

Previously, it was reported that FOXA2 phosphorylation leads to ubiquitination and degradation of FOXA2 [44]. We have previously shown that PCN triggers FOXA2 ubiquitination and degradation [28]. We examined whether Lys259 deacetylation promotes FOXA2 degradation through the ubiquitination-mediated proteasomal degradation in PCN-exposed 16HBE14o^−^ cells. Changes in endogenous FOXA2 protein abundance in 16HBE14o^−^ cells were examined in the presence of MG132, a 26S proteasome inhibitor [44]. In the absence of PCN treatment, MG132 increased the abundance of endogenous FOXA2 in a dose-dependent manner (Figure 7A) and partially rescued endogenous FOXA2 from PCN-mediated protein depletion (Figure 7B), suggesting PCN induces FOXA2 degradation by the ubiquitin-proteasome system. Given that Thr156 phosphorylation triggers FOXA2 inactivation and nuclear exclusion [42,43], we investigated whether this phosphorylation mediates FOXA2 degradation through ubiquitination. While FOXA2-WT was ubiquitinated and depleted by PCN exposure, FOXA2-T156A was resistant to PCN-mediated ubiquitination and degradation (Figure 7C). These data suggest that the Thr156 phosphorylation is important for ubiquitin-mediated degradation of FOXA2 during PCN-mediated oxidative stress. Additionally, when compared to FOXA2-WT in PCN-exposed cells, FOXA2-K259Q showed reduced ubiquitination in response to PCN (Figure 7D). Furthermore, unlike FOXA2-WT, the abundance of FOXA2-K259Q protein was not markedly altered under PCN exposure (Figure 7D). Collectively, these findings suggest that Lys259 deacetylation, in coordination with phosphorylation at Thr156, are critical steps for FOXA2 inactivation and ubiquitin-mediated proteasomal degradation during goblet cell transdifferentiation and *MUC5AC* overexpression induced by PCN-imposed oxidative stress.

## 3. Discussion

**Figure 8:**
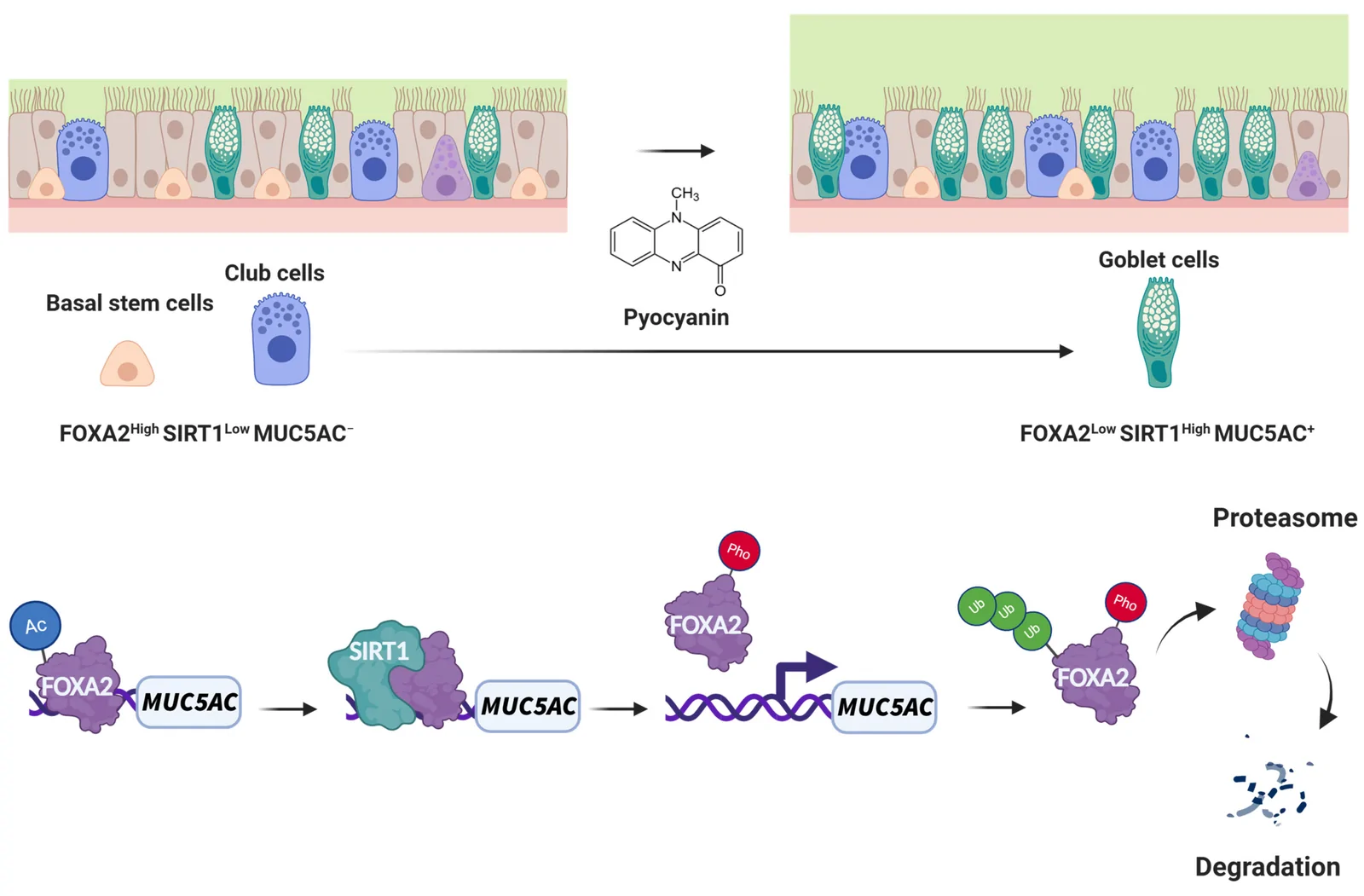
Proposed mechanism of FOXA2 inactivation by acetylation/deacetylation cycling in bronchial epithelial cells. In unstimulated bronchial epithelial cells, FOXA2 is primarily localized in the nucleus and prevents *MUC5AC* gene expression by sequestering the promoter. When the cells are exposed to PCN, SIRT1 translocates into the nucleus and inactivates FOXA2 by deacetylation at Lys259, followed by AKT-mediated phosphorylation at Thr156, leading to nuclear exclusion, ubiquitination, and proteasomal degradation.

FOXA2 is a pivotal regulator of lung morphogenesis that determines respiratory epithelial cell fates [38,39]. Previous studies have established that *P. aeruginosa* PCN depletes FOXA2 via the EGFR-AKT signaling pathway [29,32], which triggers its phosphorylation, nuclear exclusion, and cytoplasmic translocation, thereby driving goblet cell transdifferentiation and excessive gel-forming mucin biosynthesis. Herein, we show that SIRT1-mediated deacetylation at Lys259 promotes FOXA2 degradation by enhancing its phosphorylation at Thr156, which triggers *MUC5AC* expression in PCN-exposed bronchial epithelial cells (Figure 8). These findings suggest that the SIRT1-FOXA2 axis is a critical regulatory checkpoint for proceeding to goblet cell transdifferentiation and mucin overexpression in PCN-exposed airway epithelium.

SIRT1 exerts protective roles against oxidative stress by inducing cellular defense mechanisms. Under acute oxidative stress, SIRT1 expression and activation are elevated as a compensatory response to initiate antioxidant responses by deacetylating and activating various transcription factors, including NRF2, p53, and FOXOs, that are crucial in regulating antioxidant enzyme expression and damage repair [53,54]. Specifically, deacetylation of the aforementioned transcription factors by nuclear SIRT1 is critical to elevate the expression of the manganese superoxide dismutase (MnSOD or SOD2) [55]. Similarly, our previous study has demonstrated that PCN activates NRF2 and downstream antioxidative enzymes in A549 adenocarcinoma alveolar type II-like epithelial cells [33], implying concomitant activation of SIRT1 by PCN-induced oxidative stress, which is required to activate NRF2.

Although it is largely unknown how PCN activates SIRT1, previous studies in the context of mitochondrial-regulated energy metabolism suggest liver kinase B1 (LKB1) and 5′-AMP-activated protein kinase (AMPK) may be the central machinery for SIRT1 activation. Mechanistically, ATP and NAD^+^ deprivation by PCN-mediated persistent oxidative stress [51,56] may trigger LKB1 and AMPK-mediated SIRT1 activation by direct interaction or by AMPK-mediated restoration in NAD^+^ levels, respectively [52,57]. AMPK senses increased AMP levels and promotes the NAD^+^ salvage process by direct activation of nicotinamide phosphoribosyltransferase (NAMPT) [52], resulting in an increase in NAD^+^ levels, an essential substrate for SIRT1 activation. NAD^+^-mediated SIRT1 activation deacetylates LKB1, leading to LKB1-mediated AMPK activation [58]. Of note, LKB1 activates SIRT1 upon resveratrol treatment [57], implying that SIRT1 and LKB1 reciprocally upregulate each other. This feedback loop establishes higher SIRT1 activation levels under acute oxidative stress conditions, including PCN exposure. Our current study indicates that SIRT1 is activated in PCN-exposed bronchial epithelial cells.

Although there have been extensive studies on the regulatory effects of SIRT1 on cellular defense systems under diverse oxidative conditions, little is known about how SIRT1 modulates FOXA2-regulated airway mucus homeostasis under acute oxidative stress. The currently understood molecular mechanisms of FOXA2 inactivation center on AKT-mediated phosphorylation at Thr156. This axis has been extensively studied in hepatocytes and pancreatic β-cells, where phosphorylation at Thr156 inactivates FOXA2 transcriptional function associated with α-ketogenesis and β-oxidation in insulin signaling and lipid metabolism [43,44,59,60,61,62]. In these metabolic contexts, SIRT1-mediated deacetylation at Lys259 precedes phosphorylation at Thr156 in a sequential manner to inactivate FOXA2 in a nutrient- and insulin-dependent manner [43,44].

Among 19 lysine residues of FOXA2, SIRT1-mediated Lys259 deacetylation is a critical step that precedes FOXA2 phosphorylation at Thr156 [43]. DNA-binding forkhead domain spans amino acid 161 to 260 of FOXA2, and both Thr156 and Lys259 are located nearest to the N- and C-termini of the domain, respectively [44]. Accordingly, either phosphorylation at Thr156 or deacetylation at Lys259 could reduce the chromatin unwinding property and DNA binding affinity of FOXA2 by widening the DNA binding pocket. Previous studies have demonstrated that Lys259 deacetylation by SIRT1 promotes FOXA2 phosphorylation at Thr156, nuclear exclusion and cytoplasmic shuttling, ubiquitination, and proteasomal degradation [43,44]. Consistent with previous findings, our study shows that SIRT1-mediated deacetylation inactivates FOXA2 by promoting phosphorylation at Thr156 in PCN-exposed bronchial epithelial cells. Moreover, Lys259 deacetylation is important for phosphorylation to occur at Thr156. These findings indicate that SIRT1-mediated deacetylation is a conserved molecular mechanism to inactivate FOXA2 across various cell types and biological processes, including oxidative stress. Although the deacetylation-mimicking K259R mutation overrides the phosphorylation-deficient T156A phenotype to drive FOXA2 nuclear exclusion, whether the acetylation-mimicking K259Q mutant can similarly overcome the phosphorylation-mimicking T156D/E mutation to promote nuclear retention remains unknown. Addressing this question in future studies will provide a more comprehensive understanding of how crosstalk between these PTM events dictates FOXA2 pioneer function and transcriptional activity. Furthermore, comparative analyses combining various FOXA2 mutants with chromatin immunoprecipitation sequencing (ChIP-seq) and assay for transposase-accessible chromatin using sequencing (ATAC-seq) will provide clear evidence of how K259 deacetylation and Thr156 phosphorylation interact to regulate FOXA2 dynamic chromatin binding and activity.

Once phosphorylated, FOXA2 is translocated into the cytosol and subsequently degraded by the proteasome. SIRT1-mediated deacetylation promotes FOXA2 degradation by proteasome in mouse livers under normal feeding conditions, which is effectively reversed during fasting because of SIRT1 inactivation [43,44]. Phosphorylation releases FOXA2 from chromatin, which enables CRM (exportin1)-mediated nuclear export to cytoplasm, ubiquitination, and degradation [42,63]. Our study shows that both FOXA2-K259Q and FOXA2-T156A mutant recombinants are resistant to ubiquitination in PCN-exposed bronchial epithelial cells, while FOXA2-WT is markedly more ubiquitinated and susceptible to degradation. As the acetylation-mimicking mutation impedes phosphorylation, both FOXA2-K259Q and FOXA2-T156A recombinants could evade nuclear exclusion because they are able to bind chromatin. Changes in chromatin binding affinity by deacetylated and phosphorylated FOXA2 remain to be elucidated in future studies.

Intriguingly, the detrimental role of SIRT1 during PCN-mediated oxidative stress and dysregulation of mucus homeostasis contradicts its protective role in chronically diseased COPD lungs, where SIRT1 expression is severely depleted [64,65,66]. This potential paradox can be explained by mutual regulation between FOXA2 and SPDEF. FOXA2 inactivation and depletion induce *SPDEF* transcription, which is a master regulator for goblet cell transdifferentiation [34,67,68]. SPDEF reciprocally represses basal *FOXA2* transcription that prevents FOXA2 restoration in goblet cells [67]. Because SIRT1-mediated deacetylation destabilizes SPDEF protein [69], the loss of SIRT1 in COPD leads to SPDEF hyperactivation, which could reduce *FOXA2* expression by SPDEF-mediated transcriptional repression, resulting in goblet cell hyperplasia and mucus hypersecretion.

Given that SIRT1 expression is markedly depleted in airway epithelial cells of COPD lungs, increasing the expression of SIRT1 has been considered a potential therapeutic strategy for COPD with its antioxidant and anti-inflammatory properties, which mitigate disease-associated phenotypic changes in preclinical models for chronic lung diseases [70]. In the context of oxidative stress, SIRT1 augments antioxidant defense by increasing antioxidant enzyme expression, such as GPX, SOD, and HO-1, through activation of transcription factors, including NRF2 [53] and FOXO1/3/4 [71,72], which restore redox balance in COPD. Since SIRT1 depletion persistently lowers baseline *FOXA2* levels by SPDEF-mediated transcription inhibition, an increase in SIRT1 levels may rescue basal stem cells and progenitors in diseased airway epithelium by destabilizing SPDEF and restoring *FOXA2* expression, thereby limiting goblet cell transdifferentiation in diseased airways by balancing FOXA2 and SPDEF abundance. Based on these findings, improving SIRT1 expression could be beneficial for reversing chronic pathological phenotypes and further slowing disease progression in patients with chronic muco-obstructive lung diseases. Given that both high and low SIRT1 expression promotes mucin biosynthesis, balancing SIRT1 expression level is likely important to achieve airway mucus homeostasis in *PA*-infected diseased lungs.

## 4. Materials and Methods

### 4.1. Control and COPD Human Lung Tissues

Paraffin-embedded lung blocks from deidentified control organ donors and COPD patients with stable disease without overt infection (lung #1 GOLD stage IV; lungs 2 and 3, GOLD stage III) were used. The study was performed in accordance with the Declaration of Helsinki protocols and was approved by the Institutional Review Board (IRB, approval #23201) of Partners Healthcare and Temple University.

### 4.2. Mouse Study Approval

The mouse study was conducted in strict accordance with the recommendation in the Guide for the Care and Use of Laboratory Animals of the National Institute of Health. Animal protocols associated with this study were approved by the Institutional Animal Care and Use Committee (IACUC, approval #20120 and #23132) at the University of Illinois at Urbana-Champaign.

### 4.3. Mouse Model of Pyocyanin Exposure

Six-week-old male and female C57BL6 mice were purchased from Charles River Laboratories. Mice were housed in positively ventilated microisolator cages and received autoclaved food, water, and bedding. Mice (groups of 8) were intranasally challenged with 25 µg pyocyanin (in 50 µL droplet) once daily for 3 weeks, as published [28,29,35,50,73]. At the end of 3 weeks, mice were euthanized for histological analyses. Briefly, a cannula was inserted into the trachea, and the lung was slowly instilled with 10% neutral-buffered formalin at a constant pressure (25 cm H_2_O). The trachea was ligated, and the inflated lung was immersed in 10% neutral buffered formalin for 24 h before being embedded in paraffin wax. Embedded lungs were sectioned to 5 µm thickness and stained with primary antibodies and visualized with secondary antibodies conjugated to Alexa Fluor 488 (Invitrogen, Waltham, MA, USA #A11008, 1:500 dilution) or Alexa Fluor 594 (Invitrogen #A21069, 1:500 dilution).

### 4.4. Human Bronchial Epithelial Cell Cultures

The SV40-transfromed immortalized human bronchial epithelial 16HBE14o^−^ cells were originally a gift from Dr. D.C. Gruenert (University of California, San Francisco). The cells were seeded in the TC-treated dish coated with Purecol Type I Collagen Solution (#5005-100ML, Advanced Biomatrix, Carlsbad, CA, USA), diluted to 50 µg/mL collagen-I solution in cell culture grade water (20-055-CM, Corning, Corning, NY, USA). The cells were maintained in Eagle’s Minimum Essential (MEM) Medium supplemented with 10% FBS (35011CV, Corning) and 1% Penicillin-Streptomycin (30-002-CI, Corning) at 37 °C in 5% CO_2_. After reaching 80% confluence, the 16HBE14o^−^ cells were trypsinized with Trypsin-EDTA (0.05%) (25300054, Gibco, Grand Island, NY, USA) and subcultured into the fresh collagen I-coated dishes. The cells were exposed to 5 µg/mL PCN (P0046-5MG, Sigma-Aldrich, St. Louis, MO, USA) for 24 h. For the inhibition and activation of sirtuins, 5 and 10 mM nicotinamide (N0636, Sigma-Aldrich), a pan-antagonist of sirtuins, 2 µM EX-527 (10009798, Cayman Chemical, Ann Arbor, MI, USA), a specific antagonist of SIRT1, and 2 µM SRT1720 (10011020, Cayman), a specific agonist of SIRT1, were added into the cell culture, respectively. The proteasome antagonist, MG132 (SC-201270), was purchased from Santa Cruz Biotech and used at a 5 µM concentration.

Primary human small airway epithelial cells (SAECs) were obtained from Lonza ((#CC-2547, Portsmouth, NH, USA) and cultured in submerged conditions at 37 °C in 5% CO_2_. SAECs were maintained in the PneumaCult^TM^-NGEx Medium (#100-1505) supplemented with 50× supplement and hydrocortisone (#07925) from Stem Cell Technology (Vancouver, BC, Canada). The cells were seeded onto 50 µg/mL collagen-I-coated dishes and subcultured to 80% confluence before use.

### 4.5. Real-Time qPCR Analysis

To analyze gene expression levels, total RNAs were extracted from 16HBE14o^−^ cells by using the RNeasy RNA purification mini kit (74104, Qiagen, Germantown, MD, USA) following the manufacturer’s recommendation. The purified total RNAs were used for establishing cDNA libraries by using the SuperScript III First-strand Synthesis System (18080051, Invitrogen). cDNA libraries were used for measuring the relative expression levels of *MUC5AC*, *FOXA2*, and *SIRT1* over *GAPDH* by performing qPCR with PowerUp SYBR green PCR Master Mix (A25742, Applied Biosystems, Carlsbad, CA, USA). The following are the primer sequences of target mRNAs: *MUC5AC* sense (GGAACTGTGGGGACAGCTCTT), *MUC5AC* anti-sense (GTCACATTCCTCAGCGAGGTC), *FOXA2* sense (GGAACACCACTACGCCTTCAAC), *FOXA2* anti-sense (AGTGCATCACCTGTTCGTAGGC), *SIRT1* sense (TAGACACGCTGGAACAGGTTGC), *SIRT1* anti-sense (CTCCTCGTACAGCTTCACAGTC), *GAPDH* sense (TGG GCT ACA CTG AGC ACC AG), *GAPDH* anti-sense (GGGTGTCGCTGTTGAAGTCA).

### 4.6. Generation of HA-Tagged FOXA2 Recombinants

HA-tagged *FOXA2* wildtype (*FOXA2*-WT), acetylation mimicking mutant (*FOXA2*-K25Q) and phosphorylation-deficient mutant (*FOXA2*-T156A) constructs were synthesized by Twist Bioscience. These gene constructs were subcloned into pcDNA3 plasmids by using the Gibson assembly master mix (E2611L, New England Biolabs, Ipswich, MA, USA), and the mutations were confirmed by DNA sequencing at the University of Illinois Carver Biotechnology Center DNA Services Core. The recombinant plasmids were transformed and amplified in *Escherichia coli* DH5α with carbenicillin selection and purified by using the PureLink HiPure Plasmid Maxiprep Kit (K210006, Invitrogen). 16HBE14o^−^ cells were electroporated with each FOXA2 recombinant plasmid by using the SF Cell Line 4D-Nucleofector kit (V4XC-2024, Lonza, Portsmouth, NH, USA) following the procedures from the manufacturer. Briefly, 1.0 × 10^6^ 16HBE14o^−^ cells were electroporated with 1 µg recombinant plasmids and incubated overnight. The transformed cells were selected by incubation with 500 µg/mL Geneticin. After antibiotic selection, the cells were maintained with 300 µg/mL Geneticin for further assays. The expression levels of recombinant FOXA2 proteins were measured by Western blot analysis. Briefly, 1.0 × 10^6^ 16HBE14o^−^ cells expressing wild-type and mutant-HA-fused FOXA2 were seeded in 100 mm TC-treated plates prior to harvest after 24 h incubation. To examine total expression levels of recombinant FOXA2 proteins, whole-cell lysates were extracted using M-PER^TM^ Mammalian Protein Extraction Reagent (#78501, Thermo Scientific, Waltham, MA, USA). We confirmed that recombinant FOXA2 proteins were similarly expressed across transfected cells independent of mutations, as shown in Figure 5A. Nuclear localization of recombinant FOXA2 was validated by immunofluorescence staining and Western blot analysis of nuclear protein extracts. Details of the experimental procedure are explained in the following sections: “Western blot analysis” and “Immunofluorescence staining in 16HBE14o^−^ cells”.

### 4.7. SIRT1 Knockdown by RNA Interference

Pre-designed *SIRT1* siRNA (Assay ID: 136458) and Silencer^TM^ Select Negative Control No.1 siRNA (4390843) were purchased from Invitrogen. Briefly, 1.0 × 10^6^ 16HBE14o^−^ cells were transfected with negative control or *SIRT1* siRNA by using the SF Cell Line 4D-Nucleofector L Kit (V4XC-2024, Lonza), following the manufacturer’s recommendation, and incubated for 48 h. Transfected cells were exposed to 5 µg/mL PCN for 24 h prior to nuclear protein lysate preparation. *SIRT1* siRNA knockdown efficiency was confirmed by a dose-dependent siRNA transfection. A volume of 20 nM of *SIRT1* siRNA exhibited the maximal knockdown efficiency without cytotoxicity from 48 h post-transfection in 16HBE14o^−^ cells. Transfection of scrambled control siRNA did not affect the expression of *FOXA2* and protein loading controls, including GAPDH and histone H3.

### 4.8. Western Blot Analysis

Cytosolic and nuclear proteins were purified from the airway cells cultured under indicated conditions by using the Nuclear and Cytoplasmic Extraction kit (786-182, G-Biosciences, St. Louis, MO, USA). Protein concentrations were measured by using the Pierce™ BCA Protein Assay Kits (#23335, Rockford, IL, USA). Nuclear protein lysates (20 µg) were used for Western blot analyses. The lysates were mixed with 2× Laemmli sample buffer (1610737, Bio-Rad, Hercules, CA, USA) and boiled for 5 min prior to separation in SDS-PAGE gels. Separated proteins were transferred to PVDF membranes at 100 V for 1 h by using a wet protein transfer system. The transferred membranes were blocked with 5% skim milk solution in TBST buffer for 1 h, and subsequently incubated with primary antibodies overnight at 4 °C. On day 2, the membranes were probed with HRP-conjugated secondary antibodies for 1 h at room temperature, and chemiluminescent signals were detected by using the SuperSignal West Femto Maximum Sensitivity Substrate (24096, Thermo Scientific). Primary and secondary antibodies used include: anti-HNF-3β (FOXA2) (SC-101060, Santa Cruz Biotechnology, Dallas, TX, USA), anti-phospho-FOXA2 T156 (710680, Invitrogen), anti-SIRT1 (8469S, Cell Signaling Technology, Danvers, MA, USA), anti-HA antibody (SC-7392, Santa Cruz Biotech), anti-rabbit IgG, HRP-linked antibody (7074S, Cell Signaling Technology), anti-mouse IgG, HRP-linked antibody (7076S, Cell Signaling Technology). Protein abundance in the nuclear and whole-cell lysate fractions was normalized to histone H3 and GAPDH, respectively.

### 4.9. Protein Immunoprecipitation (IP) and Co-IP

IP and co-IP assays with nuclear protein lysates were performed by using the Nuclear Complex Co-IP kit (54001, Active Motif, Carlsbad, CA, USA). Except for the protein pulldown step, both IP and co-IP samples were processed identically following the procedures provided by the manufacturer. FOXA2 proteins were pulled-down by incubating with anti-FOXA2 antibody (SC-101060, Santa Cruz Biotech). Normal mouse IgG (sc-2025, Santa Cruz Biotech) was used as a negative control, which did not pull down FOXA2. To pull down HA-tagged FOXA2 wildtype and mutant recombinants, anti-HA antibody (SC-7392, Santa Cruz Biotech) was utilized. Acetylation and phosphorylation levels of FOXA2 were assessed by probing with the anti-acetyl-lysine antibody (9411S, Cell Signaling Technology) and anti-phospho-FOXA2 T156 (710680, Invitrogen), respectively. For Figure 3A, to examine the levels of immunoprecipitated FOXA2, the blots were probed with an anti-FOXA2 antibody (22474-1-AP, Proteintech, Rosemont, IL, USA). To examine the interaction between SIRT1 and FOXA2, co-IP was performed against SIRT1 and FOXA2 complexes by pulling down with anti-SIRT1 antibody (8469S, Cell Signaling Technology), which was further probed with anti-FOXA2 antibody (22474-1-AP, Proteintech).

### 4.10. Immunofluorescence Staining in Human and Mouse Lung Sections and in 16HBE14o^−^ Cells

We performed the immunofluorescence staining of human and mouse lung sections as published [28,29,35,50,73]. Briefly, deparaffinized lung sections were unmasked by incubating in the citric acid antigen retrieval buffer, followed by blocking using the M.O.M immunodetection kit (BMK-2202, Vector Labs, Newark, CA, USA) according to the manufacturer’s recommendations. The lung sections were incubated with primary antibodies at 4 °C overnight and probed with fluorescence-conjugated secondary antibodies for 1 h at room temperature. Then, the sections were mounted with Vectashield antifade mounting medium (H-1000-10, Vector Laboratories). Primary antibodies used: anti-MUC5AC antibody (SC-21701, Santa Cruz Biotech), anti-FOXA2 antibody (22474-1-AP, Proteintech), and anti-SIRT1 antibody (9475S, Cell Signaling Technology). Fluorescence-conjugated secondary antibodies used: anti-Mouse IgG, Alexa Fluor 488 (A-21202, Invitrogen), anti-Rabbit IgG, Alexa Fluor 594 (A35754, Invitrogen).

For immunocytochemistry, 16HBE14o^−^ cells were fixed with 4% paraformaldehyde and permeabilized by 0.5% Triton X-100 solution for 15 min each at room temperature. Next, the cells were incubated with DAPI for 10 min for nuclear visualization, before incubating with a blocking solution composed of 5% donkey serum (D9663-10ML, Sigma-Aldrich) in TBST for 1 h at room temperature. Subsequently, the cells were incubated with primary antibodies at 4 °C overnight. On day 2, the primary antibodies were probed with fluorescence-conjugated secondary antibodies for 1 h at room temperature. Primary antibodies used in immunocytochemistry staining: anti-MUC5AC antibody (SC-21701, Santa Cruz Biotech), anti-FOXA2 antibody (22474-1-AP, Proteintech), anti-SIRT1 antibody (9475S, Cell Signaling Technology), and anti-HA antibody (3724S, Cell Signaling Technology). Fluorescence-conjugated secondary antibodies used: anti-Mouse IgG, Alexa Fluor 488 (A-21202, Invitrogen), anti-Rabbit IgG, Alexa Fluor 594 (A35754, Invitrogen). Fluorescence intensity of FOXA2 and MUC5AC was quantified using ImageJ (Ver.1.54g; JAVA 1.8.0_345). Each marker was quantified individually from single-channel fluorescence images. To establish a standardized baseline, threshold parameters were optimized using the PCN-exposed group, which exhibited higher non-specific background noise associated with mucin hypersecretion. The threshold cutoff was defined immediately above background/noise intensity levels to ensure specific target signal capture and was applied uniformly across experimental conditions. No significant signals were captured in the negative control groups. Region of interest (ROI) was selected based on the following criteria: (i) intact bronchial epithelial structures without mechanical damage, (ii) clearly distinguishable bronchial epithelium, and (iii) regions displaying characteristic features of goblet cell transdifferentiation and mucus hypersecretion. The PCN-exposed mouse lungs served as the baseline reference due to the marked increase in MUC5AC expression and structural remodeling observed in both PCN-exposed mice and COPD patients.

### 4.11. Proximity Ligation Assay

Physical interactions between SIRT1 and FOXA2 were examined in lung sections from PCN-exposed mice and CODP patients by using the Duolink^®^ Proximity Ligation Assay (DUO92101, Sigma-Aldrich). The lung sections were deparaffinized, and antigens were retrieved as we have previously published [28,29,35,50,73]. The remaining steps were performed following the manufacturer’s protocols. To probe FOXA2 and SIRT1, anti-FOXA2 antibody (22474-1-AP, Proteintech) and anti-SIRT1 antibody (9475S, Cell Signaling Technology) were used, respectively. MUC5AC was visualized by fluorescence-conjugated anti-MUC5AC antibody (Alexa Fluor 488) (SC-21701 AF488, Santa Cruz Biotech). For the negative control, normal rabbit IgG (#2729S, Cell Signaling Technology) and normal mouse IgG (B00620, Proteintech) were added into the lung section in combination with anti-SIRT1 and anti-FOXA2 antibodies, respectively.

### 4.12. Statistical Analysis

Statistical analysis was performed by using the unpaired Student’s *t*-test, Welch’s *t*-test, and two-way ANOVA test in GraphPad Prism (Ver 11.0.1). Data were calculated as the mean ± S.E.M. Statistical significance is represented by * *p* < 0.05; ** *p* < 0.01; *** *p* < 0.001, and **** *p* < 0.0001.

## 5. Conclusions

In conclusion, our study demonstrates that SIRT1-mediated deacetylation triggers FOXA2 inactivation and nuclear exclusion in response to PCN-induced acute oxidative stress in bronchial epithelial cells, thereby driving goblet cell transdifferentiation and mucin biosynthesis. Deacetylation at Lys259 governs FOXA2 activity and stability, ultimately dictating bronchial epithelial cell fate decisions. Future studies will decipher the dynamic crosstalk between SIRT1, FOXA2, and SPDEF, and elucidate how this regulatory network fine-tunes mucin biosynthesis in healthy and diseased airways.

## Data Availability

The original contributions presented in this study are included in the article. Further inquiries can be directed to the corresponding authors.
